# The Psychological Inflexibility in Pain Scale (PIPS) – validation, factor structure and comparison to the Chronic Pain Acceptance Questionnaire (CPAQ) and other validated measures in German chronic back pain patients

**DOI:** 10.1186/s12891-015-0641-z

**Published:** 2015-07-28

**Authors:** Antonia Barke, Jenny Riecke, Winfried Rief, Julia A. Glombiewski

**Affiliations:** Klinische Psychologie und Psychotherapie, Philipps-Universität Marburg, Gutenbergstraße 18, 35037 Marburg, Germany

**Keywords:** Chronic pain, Back pain, Questionnaire, Psychological inflexibility, German, Validation

## Abstract

**Background:**

Acceptance and Commitment Therapy (ACT) for pain offers an alternative to traditional Cognitive Behavioural Therapy (CBT) approaches. ACT focuses on the enhancement of ‘psychological flexibility’ that enables individuals to pursue their values and goals despite pain. To assess specific treatment effect or mediators and moderators of change, questionnaires measuring ACT constructs are needed.

**Methods:**

The Psychological Inflexibility in Pain Scale (PIPS) was translated into German and completed by 182 participants with chronic back pain (70.3 % women, age 51.0 ± 10.5 years). Item analyses and a confirmatory factor analysis were computed as well as correlations with the Chronic Pain Acceptance Questionnaire (CPAQ), which measures related, but slightly different ACT-related constructs, and self-reported disability, pain intensity and further pain-related questionnaires.

**Results:**

The confirmatory factor analysis reproduced the original structure with two subscales and a good fit. The internal consistencies of the subscales were Cronbach’s α = .91 (Avoidance) and α = .26 (Fusion). Average item-whole correlations of the items with the respective subscales were *r* = .71 (Avoidance) and *r* = .20 (Fusion). The highest correlations were observed for Avoidance with the CPAQ (*r* = −.81), the Tampa Scale of Kinesiophobia (*r* = .58) and the Pain Catastrophizing Scale (*r* = .56) and for Fusion with the CPAQ subscale Pain willingness (*r* = −.55). The PIPS subscale Avoidance predicted pain-related disability even after controlling for catastrophizing and fear of movement.

**Conclusions:**

The PIPS subscale Avoidance may be a valuable instrument to assess treatment processes in future RCTs. The PIPS subscale Fusion seemed more problematic in the German sample with chronic back pain. More research on the comparison between PIPS and other questionnaires assessing psychological flexibility and the usefulness of the concept ‘Fusion’ for chronic pain are needed.

**Electronic supplementary material:**

The online version of this article (doi:10.1186/s12891-015-0641-z) contains supplementary material, which is available to authorized users.

## Background

Cognitive behavioural therapy (CBT) has been the psychological treatment of choice for chronic pain for the past three decades [1]. CBT has proven to be effective in comparison to standard medical treatment but, despite the large body of research on CBT for pain, the effect sizes remain small [1] to moderate [2]. Acceptance and Commitment Therapy (ACT) for pain offers an alternative to traditional CBT approaches, originally adapted from the translational approach by Hayes et al. for severely disabled, chronic patients [3]. ACT mainly focuses on the enhancement of so-called ‘psychological flexibility’. According to this theory, psychologically flexible individuals are able to pursue their values and goals despite pain and other discomforts [3]. Psychological flexibility comprises six processes: Acceptance, Defusion, Moment-to-Moment Awareness, Self-as-Context, Values Orientation and Committed Action.

First randomised controlled trials (RCTs) on ACT have shown promising results [4] and correlative [5] and laboratory [6] research has provided evidence for some of the assumptions of ACT. To assess specific treatment effects or mediators and moderators of change, questionnaires measuring ACT processes are needed. For chronic pain, two recently developed instruments exist: the *Chronic Pain Acceptance Questionnaire* (CPAQ) [7] and the *Psychological Inflexibility in Pain Scale* (PIPS) [8]. The PIPS is a 12-item questionnaire that aims at measuring psychological inflexibility with two subscales, Avoidance of Pain (Avoidance) and Cognitive Fusion with Pain (Fusion). Avoidance measures the behavioural tendency to withdraw from planned and valued activities and social participation in response to pain or its expectation (example item: ‘I do not do things that are important to me to avoid feeling my pain’). Fusion is intended to measure the entanglement of pain-related thoughts and actual experiences, i.e. the difficulty of distancing oneself from thoughts about the pain and its possible causes (example item: ‘It is important to understand what causes my pain’). The CPAQ and PIPS set out to measure different, though closely related, constructs (the acceptance of pain vs. the – inversely formulated – psychological (in)flexibility in the context of pain). The psychometric properties of PIPS have been investigated in two studies by Wicksell et al. [8, 9]; the samples included participants with mixed pain conditions and a large sample with participants who had disorders associated with whiplash. The studies confirmed a two-factor solution and acceptable reliability and validity. Furthermore, PIPS was found to mediate the relationship between pain and disability. Psychological inflexibility measured by PIPS was the mediator of improvement in ACT for patients with chronic pain following whiplash injury [10].

A recently published validation of a Spanish version of PIPS [11] confirmed the two-factor structure and showed good psychometric properties in a sample of persons with fibromyalgia. PIPS correlated with other scales such as the *Pain Catastrophizing Scale* (PCS) [12] and the *Hospital Anxiety and Depression Scale* (HADS) [13] but showed only modest associations with pain intensity. To date, no German translation of the PIPS exists and a validation of the PIPS in a larger back pain sample is still lacking.

We developed a German version of the PIPS following international guidelines of cross-cultural adaption [14]. The purpose of the present study was to assess the psychometric properties of the German adaption of the PIPS in a chronic back pain sample and to provide further data concerning the factor structure. Additionally, we aimed to assess whether the PIPS made a unique contribution to the prediction of pain-related disability over and beyond those of established instruments.

## Method

### Translation and cross-cultural adaption

Permission to translate and validate the PIPS (English version, see also Rodero et al. [11]) was obtained from the original authors. The translation and cross-cultural adaption process followed the guidelines of Beaton and colleagues [14]. Prior to the study, the pre-final version was administered to a group of five patients suffering from chronic back pain. They provided general remarks about the questionnaire and were interviewed about potential difficulties in understanding the items. The German and the back-translated versions of the questionnaire were also sent to the original authors of the PIPS, who approved the changes that were made. The German version of the PIPS can be found in the (Additional file 1).

### Participants

Persons with chronic back pain (defined as back pain that has persisted three months or more) and German mother tongue were recruited via the internet. The survey was promoted on websites of several patient organisations and support groups for chronic pain patients in Germany as well as in an inpatient rehabilitation centre (MediClin Klinik am Hahnberg, Germany). 182 persons (54 men and 128 women) with a mean age 51.0 (±10.5 years) participated in the survey. The participants’ mean pain duration was 12.7 ± 10.9 years.

All participants provided informed consent to participate, and the study was approved by the Ethics Committee of the Department of Psychology, Philipps-University Marburg, Germany.

### Instruments

#### Psychological inflexibility towards pain

The PIPS consists of 12 items rated on a 7-point scale from 1 (never true) to 7 (always true). In the original, 8 items are related to avoidance behaviours and form the subscale Avoidance. Four items concern cognitive fusion and form the subscale Fusion.

#### Pain intensity

The current and the average pain intensity over the last four weeks were assessed with a numeric rating scale (NRS-P) from 0 (no pain) to 10 (worst pain imaginable).

#### Pain acceptance

Pain acceptance was measured using the German version of the CPAQ [7, 15]. The CPAQ consists of 20 items rated on a 7-point scale from 0 (never true) to 6 (always true). The subscale Activities Engagement assesses the extent to which the person engages in activities despite their pain (e.g. ‘There are many activities I do when I feel pain.’), whereas the subscale Pain Willingness is an inverted measure of how much the person feels the need to avoid or control pain (e.g. ‘I need to concentrate on getting rid of my pain.’).

#### Pain catastrophizing

For the assessment of pain catastrophizing, the German version of the PCS was used [16, 12]. The PCS consists of 13 items scored on a 5-point scale ranging from 0 (not at all) to 4 (all the time). The items form three subscales: The subscale Rumination concerns the inability to stop thoughts about pain (e.g., ‘When I am in pain, I keep thinking how badly I want the pain to stop’). The subscale Magnification measures the tendency to exaggerate the threatening nature of the pain (e.g., ‘When I am in pain, I become afraid that the pain will get worse’). The subscale Helplessness captures feeling unable to deal with the pain (e.g., ‘When I am in pain, I feel I cannot go on’). The total score ranges from 0–52, with higher scores indicating higher levels of pain catastrophizing. The PCS has excellent psychometric properties [17].

#### Fear of movement

Fear of movement was measured using the German version of the *Tampa Scale of Kinesiophobia* (TSK) [18, 19]. The TSK is a self-report measure of fear of movement (e.g., ‘It is really not safe for a person with a condition like mine to be physically active’) and re-injury (e.g., ‘Pain always means I have injured my body’). It consists of 17 items scored on a 4-point scale from 1 (strongly disagree) to 4 (strongly agree). The resulting scores range from 17–68, with higher scores indicating higher levels of fear. Recent studies have suggested a two-factor structure, including somatic focus and activity avoidance [20]. The TSK has good psychometric properties [4].

#### Pain-related disability

Disability was measured using the German versions of the *Pain Disability Index* (PDI) [21, 22], and the *Quebec Back Pain Disability Scale* (QBPDS) [23]. The PDI measures the extent to which pain interferes with the person’s ability to engage in everyday activities (family/home responsibilities, recreation, social activities, occupation, sexual behaviour, self-care, and life-support activity). Each of the seven areas of activity are rated on an 11-point scale from 0 (no disability) to 10 (total disability). The total score ranges from 0–70, with higher scores indicating higher pain-related disability. The PDI has very good psychometric properties [24].

The QBPDS, more specifically, measures functional disability in persons with back pain. It assesses disability related to basic daily activities and postures (bed/rest, sitting/standing, ambulation, movement, bending/stooping, and handling of heavy objects). The patients are asked to rate the difficulty of performing the respective activity on the current day, using a 6-point scale ranging from 0 (not difficult at all) to 5 (unable to do). The total score is calculated as the sum of all the items with higher scores indicating higher disability.

#### Anxiety and depression

Depression and anxiety were assessed with the German version of the HADS [25, 26]. The scale was specifically designed for a clinical population with somatic symptoms and measures depressive and anxious symptoms in the past week. The 14-items are answered on a 4-point scale with item-specific response categories. The internal consistencies for both subscales, Depression and Anxiety, were good (Cronbach's α = .80 for each subscale).

### Statistical analysis

Item analyses to determine mean item scores and standard deviations, item difficulty, and corrected item-total correlations were computed for each item. Mean inter-item correlations, mean item difficulty and internal consistency (standardized Cronbach’s α) were calculated for the whole scale and each subscale separately. In order to investigate whether the factor structure corroborated the original version, a confirmatory factor analysis was conducted. For measures of fit, we reported χ^2^ test and the χ^2^/df ratio, the root mean square error of approximation (RMSEA), the standardized root mean square residual (SRMR) and the goodness of fit index (GFI).

Pearson correlations between the scores of the PIPS subscales and age, current pain intensity, average pain intensity, months since pain onset, and the TSK, the PCS and its subscales, the PDI, the QBPDS, the CPAQ and its subscales and the HADS-D and HADS-A, were calculated. For sex and PIPS score, a point biserial correlation coefficient was computed.

In addition, hierarchical multiple regressions were calculated to assess whether the PIPS made a unique contribution to the prediction of pain-related disability (PDI, criterion) over and above firstly the TSK and secondly the CPAQ. For this we used the method of blockwise forced entry and tested the incremental gain from adding the PIPS when the TSK (or CPAQ, respectively) were already part of the model, and vice versa.

We also computed a multiple regression (Enter method) with the criterion PDI and the predictor variables sex, age, average pain intensity in the last month, PCS, TSK, HADS-A, HADS-D, and the subscales Avoidance and Fusion. Multicollinearity was assessed according to the recommendations of Menard (1995) suggesting that tolerance values below .20 should be of concern. The data were analysed using Statistica version 10 (Statsoft Inc, Tulsa, USA). For the confirmatory factor analysis, AMOS version 21 (IBM SPSS, Meadville, USA) was used.

## Results

### Item analyses and internal consistency

Standard item analyses were conducted. The item difficulties, which in the context of attitude measurement show the extent to which the items are being endorsed by the respondents, ranged from *p*_i_ = .47 (item 4) to *p*_i_ = .84 (item 9) with a mean item difficulty of *p*_i_ = .57 for Avoidance and *p*_i_ = .74 for Fusion.

The highest item-whole correlation observed was *r*_itc_ = .84 (item 8) and the lowest *r*_itc_ = .07 (item 3). In general, the item-whole correlations showed a better range for Avoidance (*r*_itc_ = .65 to *r*_itc_ = .84) than for Fusion (*r*_itc_ = .07 to *r*_itc_ = .33). This was reflected by the mean item-whole correlations for the subscales (Avoidance: mean *r*_itc_ = .71, Fusion: mean *r*_itc_ = .20) and the mean inter-item correlations (Avoidance *r* = .57, and Fusion *r* = .21). For details see Table 1. The subscales Avoidance and Fusion were correlated (*r* = .25, p < .001).

**Table 1:** Item means and standard deviations, item difficulties, item-whole correlations with the subscales and Cronbach’s Alpha for the subscales if the item was removed

Item^a^		M	SD	Difficulty	Item-whole correlation^b^	α if removed
Avoidance		32.01	11.35	.57 ^c^	.71 ^c^	-
1.	I cancel planned activities when I am in pain.	4.18	1.53	.60	.65	.90
2.	I say things like ‘I don’t have any energy’, ‘I am not well enough’. ‘I don’t have time’, ‘I don’t dare’, ‘I have too much pain’, ‘I feel bad’, or ‘I don’t feel like it’.	4.40	1.69	.63	.61	.91
4.	Because of my pain, I no longer plan for the future.	3.30	1.99	.47	.70	.90
5.	I avoid doing things when there is a risk it will hurt or make things worse.	4.28	1.78	.61	.69	.90
7.	I don’t do things that are important to me to avoid pain.	3.93	1.84	.56	.70	.90
8.	I postpone things because of my pain.	4.43	1.65	.63	.84	.89
10.	It’s not me that controls my life, it’s may pain.	3.68	2.04	.53	.72	.90
11.	I avoid planning activities because of my pain.	3.82	1.88	.55	.80	.89
Fusion		20.84	6.20	.74 ^b^	.20 ^b^	-
3.	I need to understand what is wrong in order to move on.	3.83	4.88	.55	.07	.63
6.	It is important to understand what causes my pain.	5.71	1.44	.82	.33	.11
9.	I would do almost anything to get rid of my pain.	5.85	1.51	.84	.18	.20
12.	It is important that I learn to control my pain.	5.45	1.63	.78	.23	.16

Notes: ^a^ English translation of the items from [9]; the German translations of the items are listed in the appendix ^b^ Item-whole correlations with the respective subscales. ^c^ Values in these rows denote the means for the subscale. Cronbach’s Alpha for the subscales is α = .91 for Avoidance and α = .26 for Fusion

The internal consistency for the whole scale was α = .78 and for the subscales α = .91 (Avoidance) and α = .26 (Fusion). We also investigated whether the subscales would have benefitted from omitting any item. This was not the case for Avoidance, but the subscale Fusion would have improved substantially by removing item 3 (α = .63 instead of α = .26).

### Factor structure

**Fig. 1:**
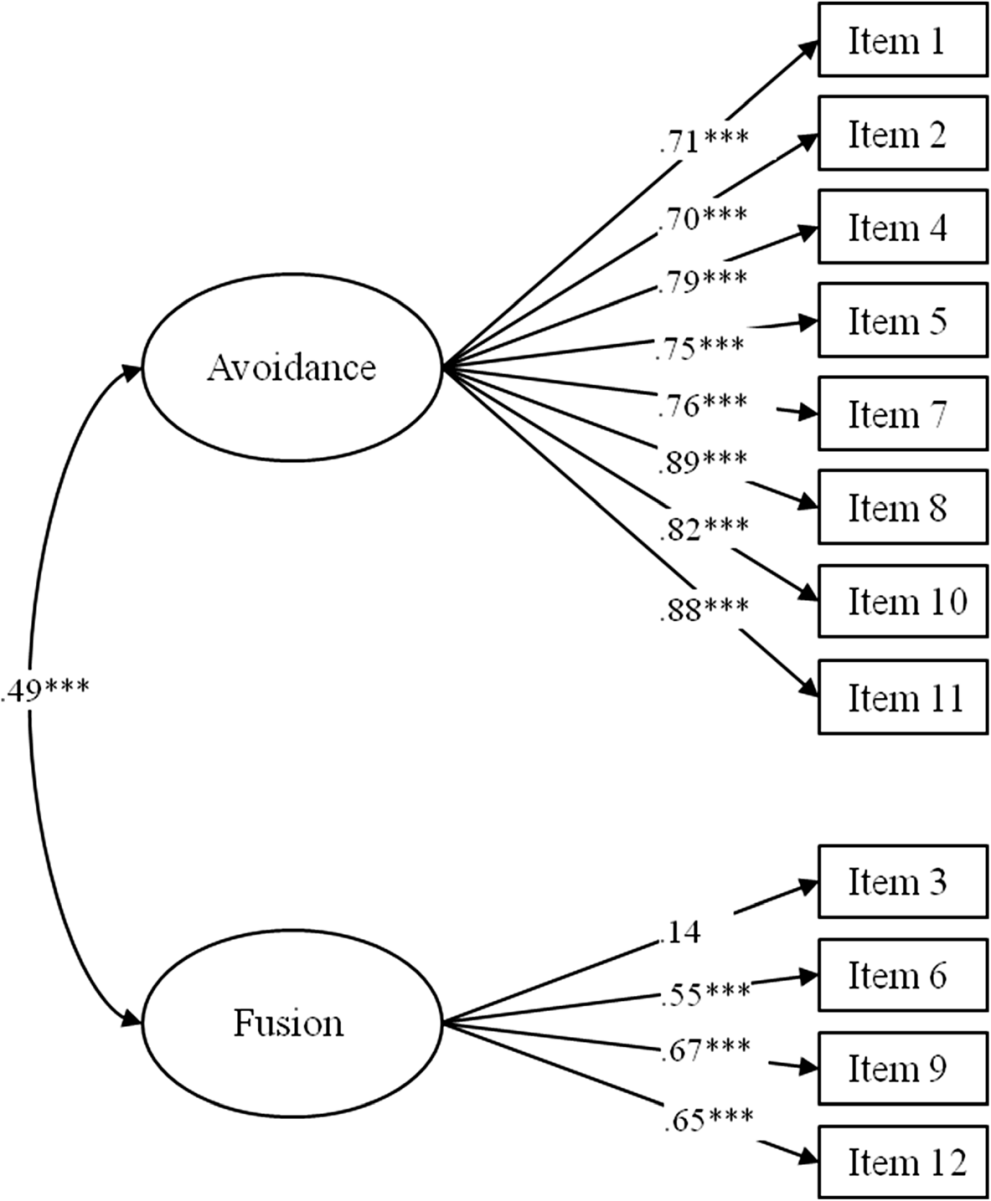
Path diagram for the confirmatory factor analysis with standardized regression weights

We tested the factor structure of the original version with a confirmatory factor analysis. The sample size for the estimation was good (ratio of n / estimated parameters = 14). All regression weights except two were at least equal to those of the original version (for the path diagram see Figure 1). The exceptions were item 3 (our model: β = .14, original: β = .62) and item 6 (our model: β = .55, original: β = .62). The overall model fit was good: χ^2^ = 81.07, df = 53, *p* = .008 with a ratio of χ^2^/df = 1.53. According to Schreiber and colleagues, a ratio of less than 2 indicates good model fit [27]. The RMSEA = .054 [CI .028 - .077] also pointed to a good fit, as values of < .08 constitute good model fit for the present sample size [28, 27]. The SRMR = .0625 was very good (Schreiber: values ≤ .08 indicate that the model should be accepted). The GFI = .925 was lower than the ideal (GFI = .95), but may still be considered acceptable.

Since both, the item analyses and the CFA, indicate a problem with item 3, this item was removed from the subscale Fusion for the further analyses.

### Correlations with age, sex and pain-related measures

*Age and sex*. Neither subscale was associated with the respondents’ sex. A small negative correlation was found between age and Avoidance (*r* = −.17, *p* < .05), but not with Fusion (see Table 2).

**Table 2:** Means and standard deviations for age and pain-related constructs and their correlations with the subscales Avoidance and Fusion (without item 3)

	M	SD	Correlation with Avoidance	Correlation with Fusion
Sex	–	–	.05	.03
Age	51.01	10.47	–.17*	–.10
Pain duration (years)	12.71	10.86	–.21*	–.26**
Current pain^a^	4.90	2.41	.24**	.06
Average pain last 4 weeks^a^	5.50	2.07	.22**	.17*
TSK	36.04	7.85	.58***	.27***
PCS	19.73	12.06	.56***	.41***
Rumination	6.63	4.14	.52***	.42***
Magnification	3.58	2.68	.46***	.42***
Helplessness	9.52	6.23	.52***	.32***
PDI	34.21	14.65	.46***	.31***
QBPDS	39.97	20.11	.42***	.14
CPAQ total	60.34	20.75	–.81***	–.43***
Activity Engagement	34.11	12.67	–.69***	–.22**
Pain Willingness	26.23	10.76	–.74***	–.55***
HADS Anxiety	10.01	3.46	.37***	.22**
HADS Depression	10.22	3.86	.48***	.12

TSK: Tampa Scale of Kinesiophobia; PCS: Pain Catastrophizing Scale; PDI: Pain Disability Index; QBPDS: Quebec Back Pain Disability Scale; CPAQ: Chronic Pain Acceptance Questionnaire; HADS: Hospital Anxiety and Depression Scale

Notes: ^a^ Pain rated on a 0–10 numeric rating scale by the person. * *p* < .05, ** *p* < .01, *** *p* < .001

*Attitudes towards pain*. The PIPS Avoidance subscale showed a high negative correlation (*r* = −.81, *p* < .001) with the CPAQ, which measures pain acceptance, and its subscales and positive correlations with the TSK, which measures avoidance beliefs regarding physical activity (*r* = .58, *p* < .001), and pain catastrophising as assessed by the PCS (*r* = .56, *p* < .001). Fusion was negatively associated with the CPAQ subscales Pain Willingness, (*r* = −.55, *p* < .001) and Activity Engagement (*r* = .22, *p* < .01) and showed positive correlations with the PCS (*r* = .41, *p* < .001) and the TSK (*r* = .27, *p* < .001).

*Self-reported disability*. PIPS Avoidance correlated moderately with self-reported disability when assessed with the PDI (*r* = .46, *p* < .001) and the QBPDS (*r* = .42, *p* < 001). PIPS Fusion correlated with the PDI ( r = .31, p < .001), but not with the QBDPS.

*Pain intensity*. PIPS Avoidance showed small correlations with current and average pain (*r* = .23 and *r* = .24, *p* < .01, respectively), whereas the Fusion subscale only correlated with average pain (*r* = .17, *p* < .05).

*Anxiety and depression*. In addition, PIPS Avoidance was associated with depressive symptoms (*r* = .48, *p* < .001) and anxiety (*r* = .37, *p* < .001) as measured with the HADS; the Fusion subscale showed a small correlation with HADS-A (*r* = .22, *p* < .001).

### PIPS and established instruments (TSK and CPAQ)

In order to test whether the PIPS made a distinct contribution to the prediction of pain-related disability after age, sex, PCS and TSK (or CPAQ, respectively) were already entered, we calculated several hierarchical regressions with blockwise forced entry.

*TSK*. When age, sex, the PCS and the TSK were already part of the model, the addition of the PIPS still improved the explained variance (ΔR^2^ = .038, *p* < .01), whereas the reverse was not true (ΔR^2^ = .006, n.s.). See Tables 3 and 4 for the full and restricted model.

**Table 3:** Multiple hierarchical regression with blockwise entry with PIPS-Avoidance and PIPS-Fusion entered in the last block

	B	SE B	β	ΔR^2^
Step 1				.040*
Constant	36.246	7.234	−	
Age	−0.186	0.105	−.132	
Sex	4.358	2.424	.134	
Step 2				.310***
Constant	14.783	8.501	−	
Age	−0.019	0.090	−.014	
Sex	3.930	2.056	.121	
PCS	0.692	0.087	.570***	
TSK	0.000	0.139	.000	
Step 3				.038**
Constant	22.476	8.864	−	
Age	−0.024	0.087	−.017	
Sex	2.673	2.044	.082	
PCS	0.635	0.094	.523***	
TSK	−0.195	0.151	−.102	
PIPS-Avoidance	0.334	0.108	.254**	
PIPS-Fusion	−0.457	0.282	−.108	

PCS: Pain Catastrophizing Scale; TSK: Tampa Scale of Kinesiophobia; PIPS: Psychological Inflexibility in Pain Scale (Avoidance: Subscale Avoidance; Fusion: Subscale Fusion without item 3)

Notes: corrected R^2^ (full model) = .366; * p < .05, ** *p* < .01, *** *p* < .001

**Table 4:** Multiple hierarchical regression with blockwise entry with TSK entered in the last block

	B	SE B	β	ΔR^2^
Step 1				.040*
Constant	36.246	7.234	−	
Age	−0.186	0.105	−.132	
Sex	4.358	2.424	.134	
Step 2				.342***
Constant	12.032	7.236	−	
Age	−0.009	0.087	−.006	
Sex	3.391	1.970	.104	
PCS	0.606	0.092	.499***	
PIPS-Avoidance	0.273	0.097	.207**	
PIPS-Fusion	−0.453	0.282	−.107	
Step 3				.006
Constant	22.476	9.240	−	
Age	−0.024	0.087	−.017	
Sex	2.673	2.044	.082	
PCS	0.635	0.094	.523***	
PIPS-Avoidance	0.334	0.108	.254**	
PIPS-Fusion	−0.457	0.282	−.108	
TSK	−0.195	0.151	−.102	

PCS: Pain Catastrophizing Scale; TSK: Tampa Scale of Kinesiophobia; PIPS: Psychological Inflexibility in Pain Scale (Avoidance: Subscale Avoidance; Fusion: Subscale Fusion without item 3)

Notes: corrected R^2^ (full model) = .366; *p < .05, **p < .01, ***p < .001

*CPAQ*. When age, sex, the PCS and the CPAQ were already part of the model, the addition of the PIPS did not significantly add to the model (ΔR^2^ = .017, n.s.). Reversely, entering first the PIPS and then the CPAQ also failed to yield an improvement over the restricted model (ΔR^2^ = .003, n.s.). See Tables 5 and 6 for the full and restricted models. Multicollinearity was assessed and did not present a problem (all tolerances > .02).

**Table 5:** Multiple hierarchical regression with blockwise entry with PIPS-Avoidance and PIPS-Fusion entered in the last block

	B	SE B	β	ΔR^2^
Step 1				.040*
Constant	36.246	7.234	−	
Age	−0.186	0.105	−.132	
Sex	4.358	2.424	.134	
Step 2				.328***
Constant	24.864	7.809	−	
Age	−0.010	0.088	−.007	
Sex	3.788	1.980	.116	
PCS	0.558	0.096	.459***	
CPAQ	−0.126	0.057	−.174*	
Step 3				.017
Constant	24.005	11.782	−	
Age	−0.008	0.087	−.006	
Sex	3.414	1.971	.105	
PCS	0.576	0.098	.474***	
CPAQ	−0.071	0.079	−.099	
PIPS-Avoidance	0.192	0.132	.146	
PIPS-Fusion	−0.490	0.286	−.116	

PCS: Pain Catastrophizing Scale; CPAQ: Chronic Pain Acceptance Questionnaire; PIPS: Psychological Inflexibility in Pain Scale (Avoidance: Subscale Avoidance; Fusion: Subscale Fusion without item 3)

Notes: corrected R^2^ (full model) = .385; *p < .05, ***p < .001

**Table 6:** Multiple hierarchical regression with blockwise entry with CPAQ entered in the last block

	B	SE B	β	ΔR^2^
Step 1				.040*
Constant	36.246	7.234	−	
Age	−0.186	0.105	−.132	
Sex	4.358	2.424	.134	
Step 2				.342***
Constant	15.939	7.734	−	
Age	−0.009	0.087	−.006	
Sex	3.391	1.970	.104	
PCS	0.606	0.092	.499***	
PIPS-Avoidance	0.273	0.097	.207**	
PIPS-Fusion	−0.453	0.282	− .107	
Step 3				.003
Constant	24.005	11.782	−	
Age	−0.008	0.087	−.006	
Sex	3.414	1.971	.105	
PCS	0.576	0.098	.474***	
PIPS-Avoidance	0.192	0.132	.146	
PIPS-Fusion	−0.490	0.286	−.116	
CPAQ	−0.071	0.079	−.099	

PCS: Pain Catastrophizing Scale; CPAQ: Chronic Pain Acceptance Questionnaire; PIPS: Psychological Inflexibility in Pain Scale (Avoidance: Subscale Avoidance; Fusion: Subscale Fusion without item 3)

Notes: corrected R^2^ (full model) = .385; * p < .05, ** *p* < .01, *** *p* < .001

### The contribution of PIPS in the prediction of pain-related disability

We also calculated a multiple linear regression for pain-related disability with the predictors age, sex, average pain intensity in the last four weeks, the PCS, the TSK, the HADS-A, HADS-D and the PIPS subscales. The PCS (β = .41, *p* < .001), the average pain intensity (β = .27, *p* < .001), and the PIPS-Avoid (β = .24, *p* < .05) contributed to the model (See Table 7) and together explained 43 % of the variance. All tolerances were well above .02.

**Table 7:** Multiple linear regression with pain-related disability (PDI) as the criterion

	M	SD	B	SE B	β
Constant	–	–	19.00	8.94	–
Sex	–	–	1.43	1.97	.04
Age (years)	51.0	10.5	−0.07	0.08	-.05
Average Pain (0–10)	5.5	2.1	1.93	0.44	.27***
PCS	19.7	12.1	0.50	0.10	.41***
TSK	35.9	7.7	−0.19	0.14	-.10
HADS-A	10.0	3.5	−0.48	0.31	-.11
HADS-D	10.2	3.9	0.41	0.29	.11
PIPS-Avoidance	31.7	11.2	0.31	0.11	.24**
PIPS-Fusion	17.0	3.5	−0.40	0.27	-.09

PCS: Pain Catastrophizing Scale; TSK: Tampa Scale of Kinesiophobia; HADS: Hospital Anxiety and Depression Scale (A: Subscale Anxiety; D: Subscale Depression); PIPS: Psychological Inflexibility in Pain Scale (Avoidance: Subscale Avoidance; Fusion: Subscale Fusion without item 3)

Notes: corrected R^2^ = .428; ** *p* < .01, *** *p* < .001

## Discussion

This is the first study presenting a German version of PIPS and investigating its psychometric properties in a sample of individuals with low-back pain. The confirmatory factor analysis demonstrated a good fit and confirmed the factor structure of the original (all but two of the regression weights were the same or better than the original), although the subscale Fusion exhibited some problems.

Regarding the psychometric investigations, the PIPS showed good item-analytic properties. Most items were of medium difficulty (between .20 and .80). Medium difficulty is psychometrically ideal because items that command very low or very high endorsement are of little diagnostic value since they fail to differentiate between the respondents. For the subscale Avoidance, the correlations of the individual items with the subscale were high and the subscale also had excellent internal consistency. Omitting any items would not have led to an improvement. The Fusion subscale seemed more problematic in the German sample. This seems largely due to item 3, which failed to correlate with the Fusion subscale. This was also reflected in the low regression weight accorded to this item in the CFA. In a model with good overall fit, item 3 was the only item that deviated markedly from the weights of the original and failed to reach significance. Interestingly, this item showed by far the largest standard deviation (4.8; by contrast the mean SD of all other items was 1.7 and the highest SD when disregarding item 3 was item 10 with SD = 2.0). Possibly, the large SD indicates that different respondents understood the item in different ways such that it failed to reliably measure the intended aspect of fusion. One possible explanation for this could lie in a problem with this item’s wording (‘I need to understand what is wrong in order to move on’). In contrast to the other items in the Fusion subscale, the item does not refer to pain or the person (‘wrong’ instead of ‘wrong with me’). The item seems to have worked in the original despite its ambiguity, but the German translation is perhaps less idiomatic, and thus it could have been the case that some of our participants took the item to be a general statement rather than a statement referring to their pain. Removing the item led to a marked improvement in the psychometric properties of the subscale: α = .63, the mean inter-item correlation reached .36 and the mean item-whole correlation = .44. We therefore excluded the item from the subscale when calculating the correlations with other measures and the regression analyses. It should be noted that without item 3 the internal consistency of the subscale – even taking into account its brevity – still leaves something to be desired.

In concordance with recent theories [29], the subscale Avoidance showed only small correlations with pain intensity but moderate to high correlations with depression and disability. Even if considered in the context of established constructs, such as fear of movement (measured with TSK) and pain catastrophizing (measured with PCS), the PIPS subscale Avoidance made a unique contribution to the prediction of pain-related disability. This result was in line with previous research [9].

The subscale Fusion, by contrast, did not predict pain-related disability. Wicksell and others [8] also found that the Avoidance subscale contributed more than the subscale Fusion to the prediction of disability in chronic pain. The authors explained this by the difficulty of measuring cognitive fusion solely with self-report questionnaires. As mentioned above, it is also possible that - despite adhering to established guidelines - the translation of the scale was problematic. An alternative explanation would be that the construct ‘Fusion’ is less important for understanding the psychological impact of chronic pain. Notably, the correlations of the subscale Fusion and the subscale Avoidance were small, indicating that these two constructs were less related than the subscale Avoidance and the CPAQ.

Our study also compared the CPAQ and the PIPS concerning their respective contributions to the prediction of pain-related disability. CPAQ and PIPS claim to measure slightly different constructs (acceptance of pain vs. psychological (in)flexibility in the context of pain). However, these constructs were strongly related with each other with acceptance being one aspect (‘process’) of psychological flexibility. Our analyses indicated that, for chronic back pain patients, the PIPS subscale Avoidance and the CPAQ were exchangeable regarding their contributions to the prediction of pain-related disability. This is understandable in light of the high correlation between CPAQ and PIPS subscale Avoidance (*r* = −.81), indicating a substantial overlap between these two questionnaires. This result was contrary to the study by Rodero et al. [11] who found that PIPS and CAPQ did not overlap. However, the participants in the study by Rodero et al. were mostly female fibromyalgia patients, whereas our sample consisted of individuals with chronic back pain and included more male participants. One explanation for the difference in the respective overlap of the questionnaires would be that acceptance of pain and psychological (in)flexibility overlap less in persons with fibromyalgia than in those with low back pain. However, since the comparability of the two studies is limited by the facts that we chose a different statistical approach by controlling for age, sex, TSK and PCS scores whereas Rodero et al. controlled only for age and pain intensity and the fact that their study was conducted in Spanish and our study in German. The latter may have led to slightly different interpretations of some items. In addition, we cannot exclude the possibility that cultural differences between Spain and Germany influenced the results. Therefore the unique features of the CPAQ and the PIPS should be addressed in further research.

The current study has a number of limitations. The correlational approach did not permit drawing conclusions about whether avoidance leads to an increased pain-related disability or whether a higher degree of disability leads to more avoidance. Disability was assessed using only self-report measures. Strengths included the translation and cross-cultural adaption process following the guidelines of Beaton and colleagues [14] and controlling for several established constructs allowing us to draw conclusions about the additional utility of the PIPS to those established measures.

## Conclusion

In conclusion, the translated version of PIPS proved to be a valid and reliable measure for avoidance related to chronic pain, but showed less satisfactory psychometric qualities with regard to the subscale Fusion. The two-factor solution was confirmed. The PIPS subscale Avoidance significantly predicted pain-related disability even after controlling for catastrophizing and fear of movement. Avoidance was closely linked with functional disability and since its reduction is a main goal of psychological therapies such as CBT, ACT or exposure-based pain treatment, the PIPS subscale Avoidance may be a valuable instrument to assess treatment processes in future RCTs.

The PIPS subscale Fusion seemed more problematic in the German sample with chronic low back pain. We recommend omitting item 3 for better results and using only the subscale Avoidance if a brief measure is needed. More research on the comparison between CPAQ and PIPS and the usefulness of the concept ‘fusion’ for chronic pain is needed.

## Additional file

Additional file 1:
**The German version of the PIPS.** (DOCX 11 kb)

## Abbreviations

ACT: Acceptance and commitment therapy
CBT: Cognitive behavioural therapy
CPAQ: Chronic Pain Acceptance Questionnaire
GFI: Goodness of fit index
HADS: Hospital Anxiety and Depression Scale
PDI: Pain Disability Index
PIPS: Psychological Inflexibility in Pain scale
QBPDS: Quebec Back Pain Disability Scale
RCT: Randomized controlled trial
RMSEA: Root mean square error of approximation
SRMR: Standardized root mean square residual
TSK: Tampa scale of kinesiophobia
