# Supplementary material for: The Psychological Inflexibility in Pain Scale (PIPS) – validation, factor structure and comparison to the Chronic Pain Acceptance Questionnaire (CPAQ) and other validated measures in German chronic back pain patients
Source: BMC Musculoskelet Disord. 2015 Jul 28;16:171. doi: 10.1186/s12891-015-0641-z (PMC4517641; doi:10.1186/s12891-015-0641-z)
Supplement: Additional file 1: — The German version of the PIPS. (DOCX 11 kb) [file 12891_2015_641_MOESM1_ESM.docx]

PIPS

German Instruction:

Im Folgenden finden Sie eine Liste mit Aussagen. Bitte schätzen Sie ein für wie wahr sie die jeweilige Aussage im Moment halten, indem Sie eine der nebenstehenden Zahlen umkreisen.

German rating scale:

1 = Niemals wahr

2 = Sehr selten wahr

3 = Selten wahr

4 = Manchmal wahr

5 = Häufig wahr

6 = Fast immer wahr

7 = Immer wahr

1. Ich breche geplante Aktivitäten ab, wenn ich Schmerzen habe.

2. Ich sage Dinge wie: „Ich habe keine Energie.’, „Es geht mir nicht gut genug.’, „Ich habe keine Zeit.’, „Ich habe zu starke Schmerzen.’, „Ich fühle mich zu schlecht.’ Oder „Ich fühle mich nicht danach.’

3. Ich muss verstehen was falsch ist, um weiterzumachen.

4. Wegen meiner Schmerzen plane ich nicht mehr für die Zukunft.

5. Ich vermeide es, Dinge zu tun, wenn das Risiko besteht, dass es schmerzen wird oder zu einer Verschlechterung führt.

6. Es ist wichtig die Ursachen meiner Schmerzen zu verstehen.

7. Ich tue Dinge die wichtig für mich sind nicht, um Schmerzen zu vermeiden.

8. Ich verschiebe Dinge wegen meiner Schmerzen.

9. Ich würde fast alles tun, um meine Schmerzen loszuwerden.

10. Nicht ich kontrolliere mein Leben, sondern meine Schmerzen.

11. Wegen meiner Schmerzen vermeide ich es Aktivitäten zu planen.

12. Es ist wichtig, dass ich lerne meine Schmerzen zu kontrollieren.
